# Changing roles of universities in the era of SDGs: rising up to the global challenge through institutionalising partnerships with governments and communities

**DOI:** 10.1186/s12961-018-0318-9

**Published:** 2018-05-09

**Authors:** Fadi El-Jardali, Nour Ataya, Racha Fadlallah

**Affiliations:** 1 Department of Health Management and Policy, Faculty of Health Sciences, American University of Beirut, Beirut, Lebanon; 2 Knowledge to Policy (K2P) Center, American University of Beirut, Beirut, Lebanon; 3 Center for Systematic Reviews on Health Policy and Systems Research (SPARK), American University of Beirut, Beirut, Lebanon

**Keywords:** Sustainable development goals, universities, institutionalisation, government, community, partnerships, government–academia, cross-sectoral collaboration

## Abstract

The 2030 Agenda for Sustainable Development covers a wide range of interrelated goals, including poverty eradication and economic growth, social inclusion, environmental sustainability and peace for all people by 2030. Policy decisions to meet the Sustainable Development Goals (SDGs) need to be informed by policy-relevant evidence co-designed and co-produced with the pertinent stakeholders, taking into consideration local and political contexts. Universities are uniquely placed to lead the cross-sectoral implementation of the SDGs and advance the 2030 agenda. This commentary provides the case for building, strengthening and institutionalising university partnerships with governments and communities to achieve the SDGs. The authors call for a change in mindsets and culture in both academia and government, and invite both parties to start the dialogue if we are to rise up to the global challenge.

## Background

The 2030 Agenda for Sustainable Development puts forward a broad and ambitious agenda covering a wide range of interrelated goals, including poverty eradication and economic growth, social inclusion, environmental sustainability and peace for all people by 2030. An analysis of the Sustainable Development Goals (SDGs) demonstrates the interconnectedness of the goals and targets, with various nexuses identified among sectors, such as the education, gender and health nexus; the energy, food security and poverty eradication nexus; the water, energy and food nexus; and the climate, land, energy and water nexus [1]. The depth and breadth of the SDGs necessitate concerted and coordinated efforts across all sectors and actors [2, 3].

Achieving progress on the SDGs will undoubtedly require the involvement of governments to work across policy areas; however, political commitment alone will not suffice without mechanisms to steer their implementation. Policy decisions to meet the SDGs will need to be informed by policy-relevant evidence, co-designed and co-produced with the pertinent stakeholders, taking into consideration local and political context [4].

Universities are uniquely placed to lead the cross-sectoral implementation of the SDGs, providing an invaluable source of expertise in research and education on all sectors of the SDGs, in addition to being widely considered as neutral and influential players. While the focus of this commentary is on the role of universities, it is acknowledged that think tanks and other institutions involved in the production and communication of knowledge also have an important role in advancing the SDG agenda.

Worldwide, some universities have started to come on board with the SDGs, prompted by United Nations-supported initiatives such as the Higher Education Sustainability Initiative, the Principles of Responsible Management Education initiative, and the Sustainable Development Solutions Network [5]. However, the question remains as to how universities, particularly those in low- and middle-income countries (LMICs), can assume a proactive and leading role in achieving the SDGs. This is particularly relevant in light of the latest United Nations report showing that the rate of progress in many areas of the SDGs is far slower than needed to meet the targets by 2030 [2].

To help universities accelerate action on the SDGs, the Sustainable Development Solutions Network Australia/Pacific published a practical guide that provides an overview of how universities can contribute to implementing the SDGs [5]. This commentary further draws on this discussion and puts forward the centrality of university-led partnerships with governments and communities to achieve the SDGs. It first makes the case for institutionalising university partnerships with governments and communities, and then proceeds with discussing the benefits of such partnerships for achieving the SDGs.

## The case for sustainable partnerships with the government and community

Fostering partnerships with governments and communities is gaining increased prominence as the mission of universities is gradually moving beyond the tradition of education and research towards a ‘third mission’ related to their ability to partner with governments and communities to achieve societal impact [6, 7]. Increasingly, universities are engaging with renowned international institutions, governments and community members. However, with few exceptions, these interactions are often ad-hoc, short-lived (e.g. for a project) or unsustainable (e.g. based on memoranda of understanding). They are not publicised as clearly, broadly and directly as needed, with unclear portals of access for governments and communities into universities. The situation is exacerbated in LMICs, where universities are grappling with the challenges of expanding research and academic capacity and fostering quality, while maintaining equitable access and relevance to economy and policy-making [8, 9]. In many instances, governments are not aware of the large and relevant knowledge base and expertise residing within universities, and academics do not perceive governments as partners in or users of their knowledge. Consequently, the potential of each partner is not being harnessed to the fullest.

**Fig. 1:**
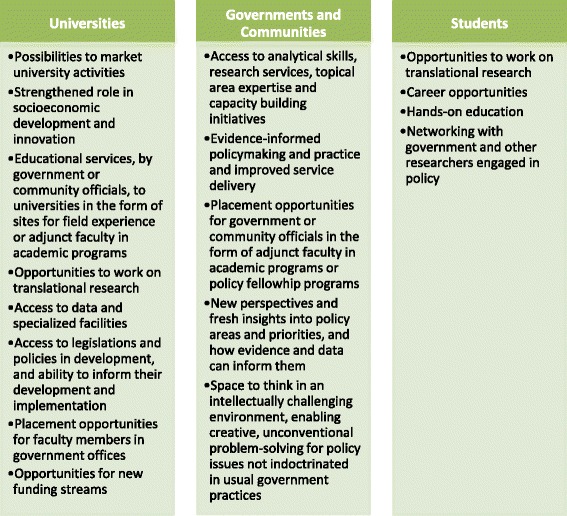
Benefits of university partnerships with governments and communities

What is needed are long-term and sustainable strategic partnerships to bring universities, governments and the communities they serve together in addressing pressing challenges and transforming societies [7, 10] (Fig. 1).

## Institutionalising partnerships with the government and community

Building long-term and sustainable partnerships is a non-trivial issue and requires investment and maintenance [11]. While the role of universities in cross-sectoral partnerships for addressing broad social challenges remains under-researched, universities will need to tailor their partnership approach to their own context and learn from their own experiences [12, 13]. Nonetheless, lessons learned from implementing cross-sectoral partnerships suggest that trust and transparency on motivations for collaboration among partners, clarity on mutual benefits, shared objectives and long-term commitments are key for effective partnerships [13, 14]. Additionally, experiences of universities worldwide, both public and private, suggest that institutionalising partnerships with the government and community helps to build transparent and sustainable relationships [15]. For example, in Uganda, cross-sectoral collaborations failed to carry the development agenda forward when long-term and institutionalised partnerships with academic institutions were not considered early on, whereas partnerships with the private sector, government and civil society led by the Uganda National Academy of Sciences – a stable and apolitical organisation of academics – has shown potential to drive coordinated action on the SDGs [14].

The SDGs provide a unique opportunity for universities and the scientific community in general to re-interpret institutional strategies and determine the structures and mechanisms needed to strengthen engagement with governments and communities. An overview of universities seeking to institutionalise engagement found that these have primarily focused on aspects of institutional structure and culture traditionally associated with sustainability, namely infrastructure, curricular reform, funding, leadership and policies [11, 15]. Community-engaged universities also manifest extensive involvement of executive leadership and embed the ‘engagement’ in institutional planning [11]. While a variety of institutional structures to support engagement have been cited, a pervasive trend among universities has been through establishing centralised entities, such as a Government and/or Community Relations Office, responsible for a whole-of-university approach to coordinating and organising relations with the government and community. Depending on institutional context, oversight of the entity responsible for engagement can be conducted by executives from both the university and the government [6]. A collaborative governance structure would enhance opportunities for exchange and integration across both portfolios – advances needed to address the SDGs. Institutional mechanisms, such as incentives and clear guidance, should support faculty engagement efforts as well as promote accountability for such engagement. For instance, some universities have adjusted their faculty review processes to take into account engagement activities [16].

Strengthened university partnerships with governments and communities can largely contribute to solving cross-sectoral and systemic health challenges. These include addressing the determinants of health, such as poverty and environmental factors, for reducing non-communicable diseases, exploring effective policies and strategies for universal health coverage in resource-limited settings, and adapting implementation strategies to national contexts [17–19]. Importantly, given the interconnectedness of health with other SDGs, strengthened partnerships can place universities in a unique position to push for incorporating health in all policies as a way to bring better integration and coherence to the SDGs. In fact, ‘health in all policies’ has been positioned as an essential tool for acting on the SDGs [20, 21].

Institutionalised partnerships within universities can facilitate engagement with governments and communities at different levels to achieve the SDGs, as described below.

### Play a lead role in strengthening the science–policy interface

Universities have the capacity to generate, translate and disseminate knowledge relevant to achieving the SDGs. They can work with policy-makers and other stakeholders to identify policy priorities/problems, assess policy options, implement solutions and evaluate policies. Importantly, they can help translate the SDGs into measurable and country-specific targets by actively matching academic capital with public policy priorities and making knowledge and resources readily available to the government and community.

Universities can engage in collaborative knowledge generation alongside other stakeholders leading to knowledge co-production or co-creation, which has the potential to increase societal impact of research through dynamic, locally adaptive partnerships, power sharing and ongoing conflict resolution [22]. They can also engage in reciprocal rotations, secondments, policy fellowships and internships with the government and community to encourage experiential learning and translational research.

### Provide neutral platforms for cross-sectoral dialogue

Universities can initiate and facilitate dialogue across multiple actors, including government, private sector, academic and scientific community, civil societies and the public. Dialogue can help ensure commitment to and strengthen implementation of the SDGs, as well as promote the political accountability needed to attain them.

### Support integrated and coherent policies and actions for SDGs

While there is a general consensus on the importance of policy coherence, efforts to achieve this tend to stall at the implementation phase. The interconnectedness of the SDGs provides an opportunity for universities to deepen understanding of implementation considerations for effective and coherent policies [3]. Universities can catalyse actions in this area by conducting analyses to identify policy coherence issues, enhancing understanding of connections and trade-offs for successful SDG implementation, and developing new metrics to facilitate integrated monitoring. Universities can also contribute to advancing the fields of systems thinking and planetary health through conducting integrated, transdisciplinary and context-specific research to strengthen understanding of managing interactions between environmental and human health. This is especially pertinent to LMICs, where weak regulation for sustainable consumption and production may have critical health consequences [3, 23, 24]. Importantly, universities can actively champion new governance mechanisms that promote cross-sectoral collaborations and policy coherence.

### Get involved in the political process

Universities can organise, synergise and coordinate lobbying and advocacy activities to influence and shape public policy, particularly with regards to the SDGs. At the same time, governments and other key players should ensure that universities are central in discussions on SDGs.

### Strengthen transdisciplinary learning and educational interactions

Universities are responsible for training and shaping the future leaders of sustainable development. By integrating the SDGs into curricula, they can provide students with the knowledge and skills needed to address them [5]. Moreover, they can establish educational programmes that emphasise interdisciplinary learning and promote multidisciplinary, systems approaches to solving the increasingly complex challenges facing societies today. For instance, achieving health-related SDGs in LMICs requires professionals proficient in designing and evaluating cross-cutting interventions within resource-constrained settings, developing innovative solutions and advocating for partnerships [19].

### Demonstrate commitment to effective engagement and impact

Universities have the capacity and capability to map, track and systematically document efforts to link research to policy and practice. They can establish meaningful frameworks and metrics for identifying, measuring and reporting on the right indicators in a valid way. Evaluating the impact of these efforts enables demonstration of commitment and progress, which are critical for learning and improvement, promoting transparency and sustaining partnerships.

## Conclusions

Three years into the SDG discussions, the pace of progress has not been adequate. There is still a clear disconnect between governments, academic institutions and other key actors. The risk of an SDG ‘fatigue’ may ultimately manifest in reverting to silo approaches to development.

To avoid this, a necessary starting point would be to steer the debate away from whether universities could transcend institutional boundaries and be part of the transformation of societies, to discussing how they should lead the latter. This would also require a shift in focus from data collection and monitoring of SDG progress to actively shaping better policies and actions in support of the SDGs. Universities need to embrace their changing roles and their unique position of influence. In parallel, governments and other partners need to acknowledge the role of research, data and knowledge in informing the SDGs, and the potential of academia to integrate different evidence ecosystems and disciplines for successful implementation of the SDGs.

A change in mindsets and culture is needed in both academia and government if we are to rise up to the global challenge. This is a call to initiate the dialogue. Let us start the conversation today so that we can achieve the SDGs by 2030.

## Abbreviations

LMICs: low- and middle- income countries
SDGs: sustainable development goals
